# Prognostic value of echocardiogram and cardiac biomarkers through three waves of COVID-19

**DOI:** 10.1016/j.jatmed.2025.05.002

**Published:** 2025-06-07

**Authors:** Yuchen Han, Grace A. Long, T.’shura Ali, Kailyn Deitz, Harideep Samanapally, Vidyulata Salunkhe, Fnu Deepti, Emma C. Huang, Jahnavi Sunkara, Katherine Cermack, Justin J. Huang, Emilia Wang, J. Tyson Davies, Maiying Kong, Jiapeng Huang

**Affiliations:** a Department of Bioinformatics and Biostatistics, School of Public Health and Information Sciences, University of Louisville, 485 E Gray St., Louisville, KY 40202, USA; b Department of Anesthesiology & Perioperative Medicine, University of Louisville, 530 S Jackson St., Louisville, KY 40202, USA; c Division of Infectious Diseases, Center of Excellence for Research in Infectious Diseases (CERID), Department of Medicine, University of Louisville, 501 E Broadway, Louisville, KY 40202, USA; d Department of Anesthesiology, Duke University, Durham, NC 27710, USA; e Department of Pharmacology & Toxicology, University of Louisville, 505 S Hancock St., Louisville, KY 40202, USA; f Department of Cardiovascular & Thoracic Surgery, University of Louisville, 201 Abraham Flexner Way, Louisville, KY 40202, USA; g Center for Integrative Environmental Health Sciences, University of Louisville, 500 S Preston St., Louisville, KY 40202, USA; h Louisville Clinical and Translational Research Center, Louisville, KY, USA

**Keywords:** COVID-19, Cardiac biomarkers, Echocardiography, SARS-CoV-2 variants, Survival analysis, Troponin

## Abstract

**Background:**

COVID-19 has had a significant impact on the scientific field. The multitude of effects this virus has on different organ systems is a continuously expanding range of study. The purpose of this study is to differentiate between the cardiac effects of the three different waves of COVID identified between March 2020- July 2022 using echocardiographic results and the cardiac biomarkers troponin and B-type natriuretic peptide (BNP). By using this information, we can evaluate cardiac impact from COVID-19, which may lead to effects on overall survival.

**Methods:**

Information was gathered through eight acute care facilities as part of a multi-center retrospective observational cohort study of patients hospitalized with confirmed SARS-CoV-2, who experienced a cardiovascular event and received an echocardiogram during admission between March 2020 and July 2022. A total of 222 participants were included and divided into 3 waves based on the highest SARS-CoV-2 strain prevalence at the time in Louisville, KY, USA. This consisted of 56 patients in the Original wave 1 (March–July 2020), 143 during the Delta wave 2 (September 2020–March 2021), and 23 during the Omicron wave 3 (January 2022-July 2022).

**Results:**

There were statistically significant differences in cardiac functions as indicated by echocardiogram and cardiac biomarkers. The Original wave not only showed the highest intensive care unit (ICU) admission rate of 73.2 %, but it also had a notable left ventricular diastolic dysfunction and the highest BNP levels. The Delta wave had the highest troponin value of 3.1 ng/mL. The left atrial volume of patients in the Omicron wave was significantly lower than in the Original and Delta waves. However, the tricuspid annular place systolic excursion for patients in the Omicron wave was significantly higher than the other two waves. In survival time models correlated with echocardiograph parameters, the Original wave showed significant correlations between left ventricular ejection fraction, left ventricular volume, right ventricular diameter, and survival. Delta wave showed significant correlation between ascending aortic size, right ventricular systolic pressure, right atrial pressure, and survival. the Omicron wave displayed significant correlation between peak troponin levels and survival.

**Conclusions:**

COVID-19 variants showed statistically significant impacts on cardiac function and biomarkers. There were significant correaltions between these echocardiographic parameters and biomarkers with survival. These cardiac differences can be used to determine overall severity of disease and provide insight into the level of clinical care indicated in COVID patients.

## Introduction

With continuously emerging SARS-CoV-2 variants, new clinical presentations and pathologies must be considered in patient care. The effects on the cardiovascular system might be very different among variants and must be carefully evaluated. For example, the Omicron variant was two to three times more transmissible than the alpha and delta variants and thus caused a greater number of new cases.^1^ There are significant differences in the Omicron wave compared to the initial two waves concerning cardiovascular outcomes, electrocardiography, and echocardiogram features. In addition, the disease severity of the Omicron variant appears to be less severe than the first two waves; however, elderly patients and those with pre-existing or new onset cardiovascular disease are at significant risk for morbidity and mortality due to the Omicron variant.^2–5^ However, cardiac complications were more prevalent in the first two waves than in the Omicron wave.^5^ Cardiac inflammation, injury, and microvascular thrombogenicity are lower in the Delta wave when compared to the Original wave.^6^ The mortality rate was lower during the Omicron wave when compared to the first two waves.^7^

Echocardiography, ECG and cardiac biomarkers are key to managing cardiovascular complications in COVID-19.^8–16^ B-type natriuretic peptide (BNP) and troponin offer a cheap and quick monitoring method. Multiple studies have researched different biomarkers in predicting outcomes of COVID-19. Thyroid dysfunction was correlated to high levels of biomarkers of inflammatory repones with cardiac injury.^17^ Prealbumin increases were seen as a risk factor associated with high-risk individuals.^18^ Multiple labs tests can be monitored to assess functionality. Studies found a significant difference in the levels of D-dimer and Troponin values in those who died or were critically ill.^19–21^ In addition, CK levels were found to be only significantly higher in mortality cases.^22^ Of all these possibilities, BNP and troponin values outperformed other routinely used biomarkers in disease severity.^23^

There has been a growing interest in markers and parameters to identify high risk patients developing heart failure in COVID-19.^2,14,24–28^ This study aims to differentiate among the cardiac effects of the three different waves of COVID between March 2020–July 2022 using echocardiographic measurements and cardiac biomarkers to determine their correlation with overall survival outcomes. The primary outcome of this study are the echocardiographic parameters and cardiac biomarkers comparison among three waves of COVID.

## Materials and methods

The patient data was collected through eight acute care facilities in Louisville, KY, USA as part of a multi-center retrospective observational cohort study of patients hospitalized with confirmed RT-PCR results of SARS-CoV-2. This information was reported through the Center for Excellence for Research in Infectious Disease (CERID) at University of Louisville, Louisville, KY, USA. CERID maintains a retrospective database for clinical research in order to further study COVID virology, clinical characteristics, and emerging patterns in a given population. Information is entered into CERID’s Research Electronic Data Capture (REDCap) database, which is in accordance with HIPPA regulations. Following IRB approval, informed consent was not required in this study due to exception. The timeline of data collection included patients admitted from March 2020 through July 2022. The patients were divided into the 3 waves of infection based on the most prevalent strain in the Kentucky, USA population: the Original wave 1 from March 2020–July 2020, followed by the delta wave two September 2020–March 2021 and the omicron wave three from January 2022–July 2022.

Study participants were limited to those 18+ within the given time parameter that tested positive for COVID-19 through RT-PCR and with a cardiovascular event receiving an echocardiogram (ECHO) during the time of admission. A total of 222 participants were included with 56 in the Original wave 1, 143 in Delta wave 2, and 23 in Omicron wave 3. Demographic information was extracted from patients’ electronic medical records (EMR), including but not limited to sex, body mass index (BMI), race, age, smoking status, comorbidities, social history, and family history (Table 1). The primary data of interest in the collection process from the EMR included ECHO parameters with relevant laboratory values such as troponin and BNP. Patients with missing laboratory values or echocardiographic parameters were excluded from analysis. Notable time values measured between the 3 waves includes length of stay of all patients admitted to the hospital and further stratified into two groups based on their survival status (i.e. survivors and non-survivors). Intensive Care Unit (ICU) length of stay (LOS) was measured as the amount of time spent in the ICU.

## Statistical analysis

Baseline continuous variables were reported as medians and interquartile ranges (IQRs) and compared using Mann-Whitney U tests. Baseline categorical data were reported as frequencies and percentages and compared using Fisher’s Exact test. Spearman rank-based correlations were calculated to examine the relationship between continuous echocardiographic parameters and troponin/BNP values. Accelerated failure models using Weibull distributions were performed to compare both survival time and hospital LOS for continuous echocardiographic parameters and troponin and BNP values. Time ratios (TRs), 95 % confidence intervals, scale parameter values, and rank-based association (RA) were reported. The rank-based association (RA) serves as a test statistic to measure the strength of the relationship between echocardiographic parameters with troponin and BNP. Patients with missing laboratory values or echocardiographic parameters were excluded from the analysis of those specific values and parameters. *P*-values of less than 0.05 were considered statistically significant. All statistical analyses were performed using R version 3.5 (R Foundation for Statistical Computing).

## Results

### Descriptive statistics of study participants

There were 56, 143, and 23 hospitalized COVID-19 patients who suffered at least one cardiovascular event during hospitalization and received ECHO in Original, Delta, and Omicron waves respectively. Patients were predominantly male and non-Hispanic white in all three waves. The median of BMI for Omicron was 26.8 (IQR: 21.9, 31.3) kg/m^2^ which was significantly lower than in Delta wave, which was 30.6 kg/m^2^ (IQR: 26.4, 36.1). 43.5 % of patients were current smokers in the Omicron wave which was significantly higher than in the Original wave (16.1 %) and Delta wave (11.2 %). Hypertension and hyperlipidemia were both significantly higher in the Delta wave than in the Original and Omicron waves.

The mortality rate of the Omicron wave (17.4 %) was significantly lower than that of the Original (42.9 %) and Delta (42 %) waves. Median hospital LOS for survivors was 11 days (IQR: 6–19 days), 10 days (IQR: 6–18 days) and 8 days (IQR: 6.5–15.5 days) for the Original, Delta, and Omicron respectively. The ICU admission rates were 73.2 % for the Original wave, 62.2 % for Delta wave and 34.8 % for Omicron wave (Table 1).

### Echocardiographic parameters

Echocardiographic Parameters of patients with at least one cardiovascular event across three waves are summarized in Table 2. The percentage of patients with left ventricular diastolic dysfunction was significantly higher in the Original wave than in Delta wave (41.1 % vs 14.7 %). Patients with severe pulmonic stenosis had a higher percentage in the Original wave than in Delta wave (28.6 % vs 9.8 %). The median of left atriam (LA) volume for patients in the Omicron wave was significantly lower than in the Original and Delta waves (39 mL, IQR: 25.9–47 vs 52 mL, IQR: 37.2–71 and 54 mL, IQR: 37.6–66). However, the median of tricuspid annular plane systolic excursion (TAPSE) for patients in the Omicron wave was significantly higher than in the Original and Delta waves (25 mm, IQR: 24–27 vs 19.5 mm, IQR: 15–22.5 and 19 mm, IQR: 15–22). The median of event-trigger peak troponin (troponin drawn within 24 h of a cardiovascular event) for patients in the Delta wave was significantly higher than the Original and Omicron waves (3.1 ng/mL, IQR: 0.4–12.4 vs 0.2 ng/mL, IQR: 0.1–1.1 and 0.1 ng/mL, IQR: 0.1–1.5). The median value of BNP for patients was significantly different across all three waves, the highest of which was in the Original wave (630 pmol/L, IQR: 126.2–1458), the second highest was in Delta wave (222.6 pmol/L, IQR: 63–785) and the lowest was in Omicron wave (55.2 pmol/L, IQR: 31.5–123.4).

Significant changes include a reduction in LV diastolic dysfunction from the Original to Delta wave (*P* = 0.004), and significant differences in LA volume (*P* = 0.017 between Original and Omicron) and BNP levels (*P* = 0.001 between Original and Omicron), indicating potential clinical characteristics changes over time. However, parameters like LV ejection fraction and RV dysfunction showed no significant differences among the waves (*P* > 0.05), suggesting stability across the study periods.

### Correlations of echocardiographic parameters with troponin and BNP

Correlations of echocardiographic parameters with event-triggered peak troponin (or peak troponin, if event-triggered peak troponin was not available), as well as combined echocardiographic parameters with BNP, are shown in Table 3. Significant correlations were found between peak troponin and LVEF in the Original and Delta waves, and between B-type natriuretic peptide and LA volume, RA volume, TAPSE for the Original and Delta waves. No statistically significant differences were observed between troponin and BNP in echocardiographic changes during the Omicron wave.

### Accelerated failure time models for survival time

The association between accelerated failure time models and survival time for echocardiographic parameters, log troponin, and log BNP are shown in Table 4.

LVEF, LV volume and right ventricle (RV) diameter were significantly associated with survival time in the Original wave. One unit increase in LVEF would extend survival time by 1.5 times (TR: 1.5, 95 % CI: 1.17–1.96, *P* = 0.002). One unit increase in LV volume would shorten survival time by 0.6 times (TR: 0.6, 95 % CI: 0.44–0.85, *P* = 0.003). One unit increase in RV diameter would shorten survival time by 0.7 times (TR: 0.7, 95 % CI: 0.49–0.97, *P* = 0.035). Ascending aorta size, RV systolic pressure (RVSP) and right atrial pressure (RAP) were significantly associated with survival time in the Delta wave. One unit increase in ascending aorta size would shorten the survival time by 0.7 times (TR: 0.7, 95 % CI: 0.49–0.93, *P* = 0.015). One unit increase in RVSP would shorten survival time by 0.8 times (TR: 0.8, 95 % CI: 0.58–0.98, *P* = 0.035). One unit increase in RAP would shorten survival time by 0.7 times (TR: 0.7, 95 % CI: 0.61–0.92, *P* = 0.006). Peak troponin was significantly associated with survival time in the Omicron wave. One unit increase in peak troponin would shorten survival time by 0.3 times (TR: 0.3, 95 % CI: 0.09–0.69, *P* = 0.007).

## Discussion

This study demonstrated a trend of decreasing cardiovascular severity from the Original SARS-CoV-2 strain to the Delta and Omicron strains. The Original and Delta waves were characterized by higher mortality rates, increased ICU admissions, and significant left ventricular dysfunction, with elevated BNP and troponin levels indicating severe myocardial injury. In contrast, the Omicron wave was associated with lower mortality, improved survival outcomes, and less pronounced cardiac effects, particularly in the form of reduced left heart dysfunction and increased right heart involvement. Of note, a trend of decreasing cardiac complications is seen in chronological order of the 3 waves. These changes reflect both the evolving nature of the virus and the growing impact of increased vaccination, hospital preparedness, and improved clinical management.

Myocardial injury marker elevation, ventricular wall thickening, pulmonary artery hypertension, and cardiac complications including acute myocardial injury, arrhythmia, and acute heart failure are more common in ICU patients with COVID-19.^29–31^ There is a distinct decreasing trend in mortality rates observed in the Omicron wave; while the Original and Delta have not only higher mortality but also higher ICU admissions with longer stays. These changes through the pandemic in ICU admissions might be explained through the increased preparedness of hospitals in being able to deal with COVID-19 patients, leading to improved outcomes for patients and abilities of non ICUs to care for COVID-19 patients through the pandemic. Another factor to lower mortality rates could be the release of the vaccine to the general public, leading to improved immune response and decreased negative effects on individuals. A study from South Africa supported that the median length of stay and mortality rate decreased among Omicron patients when compared to prior waves, consistent with our results.^25^

LV diastolic dysfunction had a higher prevalence in the Original wave. LA volume in Omicron were significantly lower and the RV systolic function for patients in Omicron were significantly higher than in the Original and Delta waves. Similar findings were reported with a strong association between increased mortality and decreased left ventricular ejection fraction, and TAPSE/pulmonary artery systolic pressure ratio.^32^ Troponin for patients in the Delta wave was significantly higher. BNP levels were the highest in the Original wave and the lowest in the Omicron wave. The Original wave had the highest mortality rates and ICU admissions as well as significant left ventricular diastolic dysfunction and the highest BNP markers. These results further indicate the increased severity of disease and cardiac effects seen early in the pandemic. Right atrial volume and left atrial volume changes were correlated to increased BNP in the Original and Delta waves. Notably, BNP has been found to have a positive correlation with radiographic features of congestion in COVID-19 patients. These factors can be used in determining medications to prevent volume overload.^33^

The key echocardiographic findings correlated with survival time are increase in LV volume, ascending aorta size, RVSP, RAP, RV diameter, troponin levels, and decrease in LVEF; which dramatically reduced the survival time of hospitalized COVID-19 patients suffering cardiovascular events. These results give insight into characteristics that could indicate increased monitoring needs in these patients with alarming echocardiographic signs. The use of these markers in predicting negative outcomes can help initiate treatment plans and guide assessments of disease progression.

LVEF was found to be abnormal in a study performed in Argentina.^34^ LA reservoir strain and LA stiffness could provide a simple, readily available tool for identifying early LV diastolic dysfunction and may direct the therapy.^35^ Another study showed that the predominant echocardiographic finding in the first and second waves of COVID-19 was left ventricular and right ventricular systolic dysfunction, among which right ventricular systolic dysfunction was more common in the Original and Delta waves.^36,37^ Similarly, our findings showed increased ventricular dysfunction through the Original and Delta waves. RV dysfunction was found to be a strong indicator to short term outcomes of disease.^38,39^ Interestingly, pulmonary hypertension has been seen to correlate with increased mortality of COVID-19 patients and appears to be reversible following recovery.^40^

During the Omicron wave, reduced ejection fraction and global systolic dysfunction were observed, while the Original and Delta waves showed preserved ejection fraction but low cardiac index. Right ventricular dysfunction, pulmonary hypertension, and tricuspid regurgitation were linked to higher 30-day mortality in the earlier waves.^41,42^ We found decreases in ejection fraction, left ventricular volume, right atrial and ventricular dysfunctions increased mortality and lead to decreases in overall survival time. During the Omicron wave, the most common feature was ventricular dysfunction and new wall motion abnormalities.^43^ Data also showed that the right ventricular abnormality remained prevalent in hospitalized patients with COVID-19 infection during the Omicron surge in New York City and was strongly and independently associated with in-hospital mortality.^44^ There were multiple reports on the correlations found between an elevated troponin in critically ill patients and mortality.^11,16,21,22,45,46^ Overall, higher BNP levels have been associated with more severe disease and mortality in COVID-19 patients.^47,48^ While BNP has confounding factors affecting its reliability, its level can be used to estimate the presence of high-grade diastolic heart failure.^49^

As part of a retrospective study, this research has inherent limitations in methodology. As the data being collected came from the EMR, bias in medical records is possible in how the data was reported out. The population from which this information was retrieved is subject to selection bias, as participants included in the study were required to be admitted to the hospital. This leads to a lower overall validity as this type of cohort tends to have more chronic health issues and longer recovery times from general illnesses. In addition, the participants of this study all came from hospitals in Louisville, Kentucky, USA which may lead to bias in geographical health characteristics. With retrospective studies, there is an increased challenge in controlling such confounders such location and comorbidities. To address these biases, multiple factors were integrated into this project, including a large population size for selection. To overcome these limitations, future studies should include multiple sites across various geographical locations and healthcare settings. Another major limitation of this study was the lack of genetic confirmation of different SARS-CoV-2 variants and these waves were purely based the timeline of COVID-19 pandemic. Genetic confirmation was not feasible at the time, as variant testing was unavailable in Louisville, KY, USA, during the study period.

## Conclusions

In conclusion, COVID-19 variants showed statistically significant impacts on cardiac function and biomarkers. There were significant correlations between these echocardiographic parameters, biomarkers and survival. These cardiac differences can be used to determine overall severity of disease and provide insight into the level of clinical care indicated in COVID patients.

## Figures and Tables

**Table 1:** Summary of demographics, medical history and outcome variables stratified by different COVID-19 waves.

Variable	Total	Original wave 1	Delta wave 2	Omicron wave 3	*P*-value
No. of patients	222	56	143	23	
**Demographics**					
Male Sex (%)	144 (64.9 %)	36 (64.3 %)	91 (63.6 %)	17 (73.9 %)	0.628
Age, median [IQR], yr	66 [58.0, 74.8]	66 [61.5, 75]	67 [57.5, 75]	63 [55,73]	0.334
Black or African American (%)	63 (28.4 %)	20 (35.7 %)	40 (28 %)	3 (13 %)	0.130
White (%)	152 (68.5 %)	35 (62.5 %)	97 (67.8 %)	20 (87 %)	0.091
Hispanic/Latino (%)	7 (3.2 %)	1 (1.8 %)	6 (4.2 %)	0 (0 %)	NA
**Medical History**					
BMI, median [IQR], kg/m^2^	30.5 [25.6, 35.9]	30.9 [25.7, 36.4]	30.6 [26.4, 36.1]	26.8 [21.9, 31.3]	0.063
Obesity (%)	100 (45.0 %)	25 (44.6 %)	68 (47.6 %)	7 (30.4 %)	0.309
Never Smoker (%)	105 (47.3 %)	28 (50 %)	69 (48.3 %)	8 (34.8 %)	0.436
Current Smoker (%)	35 (15.8 %)	9 (16.1 %)	16 (11.2 %)	10 (43.5 %)	< 0.001
Former Smoker (%)	82 (36.9 %)	19 (33.9 %)	58 (40.6 %)	5 (21.7 %)	0.192
Hypertension (%)	157 (70.7 %)	34 (60.7 %)	112 (78.3 %)	11 (47.8 %)	0.002
Hyperlipidemia (%)	105 (47.3 %)	24 (42.9 %)	76 (53.1 %)	5 (21.7 %)	0.014
Renal disease (%)	50 (22.5 %)	17 (30.4 %)	31 (21.7 %)	2 (8.7 %)	0.108
Neoplastic disease (%)	20 (9.0 %)	4 (7.1 %)	14 (9.8 %)	2 (8.7 %)	0.936
Stroke (%)	32 (14.4 %)	9 (16.1 %)	20 (14.0 %)	3 (13.0 %)	0.956
COPD (%)	54 (24.3 %)	16 (28.6 %)	33 (23.1 %)	5 (21.7 %)	0.682
Asthma (%)	13 (5.9 %)	5 (8.9 %)	7 (4.9 %)	1 (4.3 %)	0.457
Obstructive sleep apnea (%)	32 (14.4 %)	6 (10.7 %)	23 (16.1 %)	3 (13 %)	0.644
Heart Failure (%)	52 (23.4 %)	12 (21.4 %)	36 (25.2 %)	4 (17.4 %)	0.717
CAD (%)	58 (26.1 %)	14 (25.0 %)	41 (28.7 %)	3 (13.0 %)	0.296
Family history of CAD (%)	47 (21.2 %)	14 (25.0 %)	30 (21.0 %)	3 (13.0 %)	0.555
Prior myocardial infarction (%)	34 (15.3 %)	8 (14.3 %)	23 (16.1 %)	3 (13.0 %)	0.960
Prior PTCA (%)	30 (13.5 %)	7 (12.5 %)	22 (15.4 %)	1 (4.3 %)	0.395
Atrial fibrillation (%)	47 (21.2 %)	12 (21.4 %)	29 (20.3 %)	6 (26.1 %)	0.817
Prior deep vein thrombosis (%)	12 (5.4 %)	3 (5.4 %)	9 (6.3 %)	0 (0 %)	NA
History of pneumonia (%)	24 (10.8 %)	7 (12.5 %)	15 (10.5 %)	2 (8.7 %)	0.945
**Hospital related outcomes**					
ICU admission (%)	138 (62.2 %)	41 (73.2 %)	89 (62.2 %)	8 (34.8 %)	0.006
ICU length of stay, median [IQR], days	10 [4, 17.8]	11 [5,18]	10 [3,16]	11.5 [5.5, 24.2]	0.717
Hospital length of stay (all patients), median [IQR], days	12 [6,18]	12 [6,18]	12 [6,18]	8 [6,16]	0.530
Hospital length of stay (Survived patients), median [IQR], days	10 [6,18]	11 [6,19]	10 [6,18]	8 [6.5, 15.5]	0.445
Time to mortality for non-survivors, median [IQR], days	13.5 [8.8, 19.2]	12 [8.5, 14.2]	14 [9.8, 20.2]	11.5 [6, 24.2]	0.557
Mortality (%)	88 (39.6 %)	24 (42.9 %)	60 (42.0 %)	4 (17.4 %)	0.069

The percentage column (%) is calculated based on the number of patients in the specific category versus the total number of patients in the respective wave.

**Table 2:** Echocardiograph parameters and cardiac biomarkers comparison among three waves of COVID.

Variable	Original wave 1	Delta wave 2	Omicron wave 3	*P*-value	w1 vs w2	w2 vs w3	w1 vs w3
No. of patients	56	143	29				
**Echocardiographic Parameters**							
LV Ejection Fraction(%) First, median [IQR]	56 [49.5, 62.5]	56 [45.3, 61.9]	45 [36.4, 55]	0.085	0.872	0.872	0.872
LV Ejection Fraction(%) Final, median [IQR]	46.5 [40.8, 52.2]	55 [52, 65.4]	47.5 [35, 57.2]	0.435	0.598	0.365	1
LV diastolic dysfunction a, No. (%)	23 (41.1 %)	21 (14.7 %)	5 (21.7 %)	0.010	0.004	0.548	0.320
RV dysfunction b, No. (%)	9 (16.1 %)	8 (5.6 %)	1 (4.3 %)	0.095	0.062	1	0.276
LV volume, median [IQR], mL	88 [67.1, 132]	93.9 [62.5, 121.5]	NA	0.508	0.533	NA	NA
LA volume, median [IQR], mL	52 [37.2, 71]	54 [37.6, 66]	39 [25.9, 47]	0.066	0.792	0.017	0.025
RV diameter, median [IQR], cm	3.6 [3, 4.2]	3.5 [3.1, 3.7]	3.2 [3.2, 3.2]	0.926	0.969	0.828	0.715
RA volume, median [IQR], mL	35.3 [28.2, 67.9]	39.2 [26.9, 56.9]	44 [41, 52.2]	0.994	0.949	0.490	0.384
Ascending aorta size, median [IQR], cm	3 [2.7, 3.3]	3.1 [2.8, 3.4]	NA	0.314	0.353	NA	NA
Peak E-wave, median [IQR], m/s	0.8 [0.6, 1]	0.8 [0.6, 1]	0.6 [0.5, 1]	0.506	0.473	0.412	0.247
TV TAPSE, median [IQR], mm	19.5 [15, 22.5]	19 [15,22]	25 [24,27]	0.047	0.512	0.008	0.015
Estimated RVSP, median [IQR], mmHg	31.5 [27, 44.5]	36.5 [30.2, 45]	31.3 [21.6, 41.8]	0.080	0.125	0.109	0.520
Estimated RAP, median [IQR], mmHg	5 [3,10]	8 [3,10]	6.5 [3,10]	0.705	0.597	0.744	0.850
MV E′ Lateral Velocity, median [IQR], m/s	0.1 [0.1, 0.1]	0.08 [0.1, 0.1]	0.07 [0.1, 0.1]	0.275	0.164	0.418	0.168
MV E/E′ Lateral, median [IQR]	8.7 [6.4, 11.3]	9.4 [7.3, 12.1]	10.4 [9.2, 14.6]	0.673	0.495	0.379	0.353
Mitral regurgitation severity c, No. (%)	4 (7.1 %)	6 (4.2 %)	2 (8.7 %)	0.485	0.477	0.323	1
Mitral stenosis severity c, No. (%)	1 (1.8 %)	0 (0 %)	0 (0 %)	0.359	0.285	1	1
Aortic regurgitation severity, No. (%)	5 (8.9 %)	12 (8.4 %)	3 (13 %)	0.728	1	0.456	0.691
Aortic stenosis severity c, No. (%)	2 (3.6 %)	2 (1.4 %)	1 (4.3 %)	0.279	0.323	0.370	1
Tricuspid regurgitation severity c, No. (%)	7 (12.5 %)	14 (9.8 %)	3 (13 %)	0.759	0.805	0.714	1
Tricuspid stenosis severity d, No. (%)	0 (0 %)	1 (0.7 %)	0 (0 %)	1	1	1	1
Pulmonic regurgitation severity, No. (%)	16 (28.6 %)	14 (9.8 %)	2 (8.7 %)	0.020	0.010	1	0.144
Pulmonic stenosis severity c, No. (%)	1 (1.8 %)	0 (0 %)	0 (0 %)	0.359	0.285	1	1
Mild or higher pericardial effusion, No. (%)	2 (3.6 %)	1 (0.7 %)	0 (0 %)	0.421	0.199	1	1
**Laboratory Values**							
First troponin, median [IQR], ng/mL	0.1 [0, 0.2]	0.1 [0, 0.3]	0.1 [0, 0.8]	0.248	0.767	0.858	0.807
Event-related peak troponin, median [IQR], ng/mL	0.2 [0.1, 1.1]	3.1 [0.4, 12.4]	0.1 [0.1, 1.5]	0.341	0.044	0.046	0.615
BNP, median [IQR], pmol/L	630 [126.2, 1458]	222.6 [63,785]	55.2 [31.5, 123.4]	0.158	0.033	0.014	0.001

**Table 3:** Spearman’s correlation coefficients (*ρ*) of echocardiographic parameters with troponin and BNP, and their associated p-values.

	Log troponin						Log BNP					
	Original		Delta		Omicron		Original		Delta		Omicron	
	ρ	*P*-value	ρ	*P*-value	ρ	*P*-value	ρ	*P*-value	ρ	*P*-value	ρ	*P*-value
LV ejection fraction, mL	−0.223	0.116	−0.24	0.007	−0.358	0.111	−0.222	0.138	−0.323	< 0.001	−0.491	0.129
LV volume, mL	0.012	0.947	0.024	0.835	NA	NA	−0.001	0.996	0.177	0.119	NA	NA
LA volume, mL	−0.067	0.711	0.100	0.373	−0.424	0.194	0.033	0.863	0.219	0.043	−0.300	0.683
RV diameter, cm	0.105	0.561	−0.142	0.627	NA	NA	0.326	0.079	−0.121	0.693	NA	NA
RA volume, mL	0.277	0.093	0.051	0.715	−0.800	0.333	0.088	0.620	0.285	0.038	NA	NA
Ascending aorta size, cm	0.273	0.390	0.095	0.638	NA	NA	−0.068	0.862	0.162	0.438	NA	NA
Peak E-wave, m/s	0.165	0.256	−0.061	0.564	−0.164	0.650	0.255	0.091	0.184	0.078	−0.400	0.750
TV TAPSE	−0.091	0.591	−0.131	0.212	−0.6	0.350	−0.366	0.036	−0.110	0.290	−1	1
Estimated RVSP, mmHg	−0.051	0.795	0.237	0.068	0.363	0.223	−0.092	0.633	0.212	0.093	0.429	0.419
Estimated RAP, mmHg	0.222	0.223	0.197	0.109	0.364	0.182	−0.162	0.376	0.060	0.625	0.189	0.654
MV E′ Lateral Velocity, m/s	−0.127	0.459	−0.191	0.075	−0.458	0.215	0.011	0.951	−0.022	0.839	0	1
MV E/E′ Lateral	0.259	0.111	0.075	0.538	0.700	0.233	0.285	0.097	0.235	0.056	0.400	0.750

**Table 4:** Accelerated failure time models for survival time.

	TR1	Confidence interval	*P*-value w1	B1	TR2	Confidence interval	*P*-value w1 vs w2	B2	TR3	Confidence Interval	*P*-value w1 vs w3	B3
Variable												
LV ejection fraction, mL	1.5	[1.17, 1.96]	0.002	0.66	0.8	[0.68, 1]	0.052	0.72	0.5	[0.22, 1.18]	0.114	0.54
LV volume, mL	0.6	[0.44, 0.85]	0.003	0.58	1.1	[0.85, 1.51]	0.396	0.62	NA	NA	NA	NA
LA volume, mL	1.0	[0.72, 1.52]	0.821	0.71	1.0	[0.82, 1.23]	0.992	0.66	0.5	[0.1, 2.85]	0.459	0.61
RV diameter, cm	0.7	[0.49, 0.97]	0.035	0.69	0.9	[0.64, 1.17]	0.334	0.53	NA	NA	NA	NA
RA volume, mL	0.9	[0.65, 1.3]	0.637	0.80	0.9	[0.67, 1.3]	0.684	0.66	NA	NA	NA	NA
Ascending aorta size, cm	1.6	[0.77, 3.29]	0.214	0.93	0.7	[0.49, 0.93]	0.015	0.53	NA	NA	NA	NA
Peak E-wave, m/s	0.7	[0.54, 1.02]	0.064	0.71	1.1	[0.85, 1.35]	0.578	0.68	0.4	[0.03, 5.74]	0.498	0.75
TV TAPSE	1.2	[0.77, 1.87]	0.420	0.84	0.9	[0.69, 1.05]	0.136	0.75	NA	NA	NA	NA
Estimated RVSP, mmHg	1.0	[0.73, 1.27]	0.776	0.46	0.8	[0.58, 0.98]	0.035	0.75	0.3	[0, 18.49]	0.561	0.85
Estimated RAP, mmHg	0.8	[0.6, 1.14]	0.243	0.60	0.7	[0.61, 0.92]	0.006	0.68	1.6	[0.06, 41.28]	0.778	1.65
MV E′ Lateral Velocity, m/s	1.1	[0.77, 1.57]	0.615	0.66	1.1	[0.88, 1.32]	0.483	0.66	0.1	[0, 154.25]	0.549	0.50
MV E/E′ Lateral	0.8	[0.61, 1.17]	0.319	0.75	1.0	[0.77, 1.43]	0.760	0.62	0.8	[0.31, 2.3]	0.740	0.52
Troponin, log ng/mL	1.0	[0.69, 1.41]	0.952	0.75	1.1	[0.9, 1.35]	0.365	0.75	0.3	[0.09, 0.69]	0.007	0.33
BNP, log pmol/L	0.8	[0.63, 1.03]	0.087	0.60	0.9	[0.76, 1.19]	0.656	0.79	0.5	[0.19, 1.07]	0.072	0.44

* The table presents regression coefficients (B) and their corresponding confidence intervals (CI) for various echocardiographic parameters, Troponin (log ng/mL), and BNP (log pmol/L) across three groups (TR1, TR2, TR3). Significant associations were observed for RV diameter in TR1 (B = 0.7, CI = [0.49, 0.97], *P* = 0.035) and ascending aorta size in TR2 (B = 0.7, CI = [0.49, 0.93], *P* = 0.015) with p-values indicating statistical significance. Conversely, variables like LA volume, RA volume, and several others did not demonstrate significant associations across the groups, suggesting varying impacts of these parameters in different clinical contexts.

## Data Availability

The data that support the findings of this study are available from the corresponding author upon reasonable request.
